# e-CoVig: A Novel mHealth System for Remote Monitoring of Symptoms in COVID-19

**DOI:** 10.3390/s21103397

**Published:** 2021-05-13

**Authors:** Afonso Raposo, Luis Marques, Rafael Correia, Francisco Melo, João Valente, Telmo Pereira, Luis Brás Rosário, Filipe Froes, João Sanches, Hugo Plácido da Silva

**Affiliations:** 1 Instituto Superior Técnico (IST), Av. Rovisco Pais n. 1, 1049-001 Lisboa, Portugal; jose.r.c.correia@tecnico.ulisboa.pt (R.C.); francisco.de.melo@tecnico.ulisboa.pt (F.M.); 2 Instituto de Sistemas e Robótica (ISR), Av. Rovisco Pais n. 1, Torre Norte—Piso 6, 1049-001 Lisboa, Portugal; 3 BrainAnswer, Lda., Rua Engenheiro Pires Marques, Lote 61, n. 5—Dto, 6000-406 Castelo Branco, Portugal; info@brainanswer.pt (L.M.); valente@ipcb.pt (J.V.); 4 Escola Superior de Saúde Dr. Lopes Dias—Instituto Politécnico de Castelo Branco, Av. Pedro Alvares Cabral 12, 6000-084 Castelo Branco, Portugal; 5 Laboratory for Applied Health Research (LabinSaúde), Polytechnic Institute of Coimbra, Coimbra Health School, Rua 5 de Outubro—SM Bispo, Apartado 7006, 3046-854 Coimbra, Portugal; telmo@estescoimbra.pt; 6 Centro Cardiovascular da Universidade de Lisboa (CCUL), Faculdade de Medicina da Universidade de Lisboa (FMUL), Av. Prof. Egas Moniz MB, 1649-028 Lisboa, Portugal; lsrosario@medicina.ulisboa.pt; 7 Hospital Pulido Valente Intensive Care Unit, Alameda das Linhas de Torres, 117, 1769-001 Lisboa, Portugal; filipe.froes@gmail.com; 8 Instituto de Telecomunicações (IT), Av. Rovisco Pais n. 1, Torre Norte—Piso 10, 1049-001 Lisboa, Portugal

**Keywords:** COVID-19, mHealth, telemedicine, pulse oximetry, temperature

## Abstract

In 2019, a new virus, SARS-CoV-2, responsible for the COVID-19 disease, was discovered. Asymptomatic and mildly symptomatic patients were forced to quarantine and closely monitor their symptoms and vital signs, most of the time at home. This paper describes e-CoVig, a novel mHealth application, developed as an alternative to the current monitoring paradigm, where the patients are followed up by direct phone contact. The e-CoVig provides a set of functionalities for remote reporting of symptoms, vital signs, and other clinical information to the health services taking care of these patients. The application is designed to register and transmit the heart rate, blood oxygen saturation (${\mathrm{SpO}}_{2}$), body temperature, respiration, and cough. The system features a mobile application, a web/cloud platform, and a low-cost specific device to acquire the temperature and ${\mathrm{SpO}}_{2}$. The architecture of the system is flexible and can be configured for different operation conditions. Current commercial devices, such as oximeters and thermometers, can also be used and read using the optical character recognition (OCR) functionality of the system. The data acquired at the mobile application are sent automatically to the web/cloud application and made available in real-time to the medical staff, enabling the follow-up of several users simultaneously without the need for time consuming phone call interactions. The system was already tested for its feasibility and a preliminary deployment was performed on a nursing home showing promising results.

## 1. Introduction

A viral pneumonia outbreak reported in December 2019, in Wuhan, China [1,2], was caused by a new virus SARS-CoV-2 (severe acute respiratory syndrome—coronavirus 2), a member of the coronavirus family. First identified in the 1960s, this group of viruses has between 60–140 nm and 30k+ bases and is found mostly in bats, birds, and reptiles. It is the 7th lineage of the Coronavirus family [3], thought to have crossed the species barrier, and causes a human respiratory disease named COVID-19.

SARS-CoV-2 changed the world. This highly contagious pathogen spread worldwide in months and healthcare facilities were quickly overwhelmed with the burden of patients. To tackle this pandemic, three methods are being set in place: vaccination, pharmacologic therapy, and the so-called non-pharmacologic intervention (NPI), which includes lockdown, social distancing, mask-wearing, testing, and tracing. An important NPI component is the increase the symptoms tracking and vital signs monitoring capabilities, in particular for mild or asymptomatic patients at home or in isolated accommodations. Providing the tools for easy visualization and management of the collected data is also important, which would allow for a closer monitoring of these patients and a quicker clinical response if necessary.

e-CoVig is a novel highly adaptable mHealth system developed for the remote monitoring of symptoms in COVID-19 patients, which uses the mobile phone as a primary sensing device. Our approach is designed to enable rapid deployment at affordable costs and followed a “human-friendly” design approach. This system is indicated for patients diagnosed with this disease and subjects undergoing quarantine, aiding in the screening of COVID-19. The main contributions of our work are the cloud-connected mobile application for remote symptomatology monitoring, the specialized wearable device that can be deployed at scale, and a feasibility study that precedes a full scale real-world evaluation. The remainder of the work is organized as follows: Section 2 establishes the background to the problem; Section 3 describes the state-of-the-art in mHealth applications; Section 4 details the implementation of our e-CoVig approach; Section 5 summarizes the feasibility evaluation results; finally, Section 6 provides a discussion and outline of the main findings.

## 2. Background

In humans, four active CoV lineages generally account for 15-30% of common colds in children and adults (229E, NL63, OC43, and HKU1) [4]. Three other particularly aggressive forms cause respiratory disorders; designated as severe acute respiratory syndrome (SARS) and middle east respiratory syndrome (MERS), the variants are known as SARS-CoV (active between 2002–2004), MERS-CoV (active since 2012), and SARS-CoV-2 (active since 2019).

SARS-CoV-2 causes the COVID-19 disease, which ranges from mild influenza-like syndrome to a life-threatening respiratory infection. Compared with the previous pandemics (influenza-derived), COVID-19 is the first from the coronavirus family, which means that the world was much less prepared to manage it [5,6,7]. COVID-19 initially lacked specific pharmacological therapy or vaccines, and the physio-pathological process was unknown.

SARS-CoV-2 is inhaled, affecting the upper respiratory system with fever, smell perturbations, sneezing, and headaches [8]. Based on the available information [9,10], SARS-CoV-2 causes an exacerbated thrombo-inflammatory process with initial pulmonary involvement (although it quickly affects other organs), which results in hypoxemia, often without the subjects noticing [11], leading to a deficient relation between ventilation and perfusion. In the worst case, patients may need either non-invasive or invasive ventilation to enable lung oxygenation support. As much as 15–20% of the people hospitalized with COVID-19 require a ventilator, while more than 70% of patients who enter intensive care need one [12,13].

The most frequent symptoms are fever, tiredness, dry cough, myalgia, dyspnea, loss of smell, gustatory dysfunction, rhinorrhea, asthenia, and sore throat [5,14,15,16]. “Silent” hypoxemia (i.e., unperceived lack of oxygen), and persistent high fever, are among the main early physical signs. Hospital admission criteria include persistent high fever (>38 °C) for the past 48 h, increased respiratory rate (≥24 CPM), and decreased oxygen saturation (≤94%).

These parameters can be monitored preventively at home. Such monitoring can consist of a frequent recording of body temperature, oxygen saturation, respiration, photoplethysmography (PPG), heart rate (HR), audio for cough characterization, and also self-report questionnaires or other relevant clinical data for the characterization of the pathology, e.g., to assess smell or gustatory dysfunction.

Mobile health (mHealth) pertains to the use of mobile devices, such as smartphones, tablets, and related personal use devices to deliver healthcare services [17,18]. As highlighted by Gonzalez et al. [17], mHealth systems typically comprise a: (1) sensor unit; (2) mobile unit; (3) remote unit. The same authors concluded that although mHealth is a topic of growing interest, there is a lack of integrated solutions, which would enhance multiple open challenges in existing approaches. COVID-19 demanded a quick and specialized response, involving symptom tracking as well as physiological sensing.

**Figure 1:**
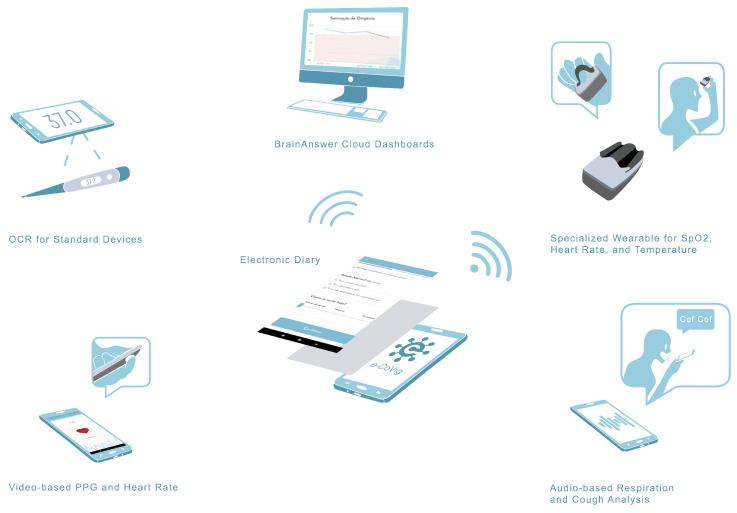
e-CoVig mHealth system overview.

Although several mHealth systems exist, we present a novel approach for at-home support and self-reporting of the outcomes using the smartphone as the primary data collection interface. Special emphasis has been given to usability, providing a “human-friendly” end-to-end solution for automated COVID-19 patient tracking (Figure 1).

## 3. State-of-the-Art

As of May 2020, more than 200 COVID-19 related applications were found on the App Store and Google Play Store [19]. These applications present various features, the most frequent being [19,20]: (a) General information about the coronavirus pandemic; (b) news; (c) recording of symptoms; (d) contact tracing apps. The main features of our approach are the patient diary, the smartphone-based physiological sensing, and the specialized device. An example of a comparable approach is the “K-note” app [21]. Developed in Japan, this application was adapted for use in the coronavirus outbreak; its main features are recording individual patient data and symptoms, and sending them via email to appropriate health professionals in a CSV file. Between March and May, the application was installed by more than 20,280 users and 400 facilities and organizations across Japan.

Other applications and platforms were developed to address the COVID-19 pandemic. In Portugal, the web-platform Trace COVID-19 was developed by the National Health System for symptom tracking and managing patients. In this platform, healthcare professionals can register specific information of infection cases, conduct contact tracing, surveillance, and clinical follow-up of patients with suspected or confirmed COVID-19. However, the information must be gathered and inserted manually into the platform by healthcare professionals, increasing the overall workload. In the United Kingdom, a health science company called ZOE, together with London King’s College launched the COVID Symptom Study app [22], which aims to gather information about COVID-19 to better understand the disease and its evolution. Users that install this application provide information regarding their symptoms daily. All this information is gathered and processed using software algorithms to improve the understanding of COVID-19 symptoms, how fast the virus is spreading in different areas, identify risk areas in the country, and better understand symptoms linked to health conditions. It is important to note that this application is not intended to be a diagnostic tool.

A major difference of previous work to our work is the goal of developing a smartphone-based platform for symptoms and vital signs monitoring, where referred individuals report their symptoms and regularly measure their vital signs, while health professionals monitor the registered information “on the other side”. Therefore, our application implements phone-based methods for signal acquisition using the smartphone built-in sensors, such as the camera or microphone. To evaluate vital signs that require specialized sensors (e.g., body temperature), a low cost wearable was developed. There are other clinical devices that measure blood oxygenation, heart rate, and body temperature, such as the JERRY-II+(AA) compact pulse oximeter, or the OXIrate hand-held pulse oximeter; however, these devices are typically expensive and difficult to integrate into other platforms. In Portugal, a group of students and researchers at Nova School of Science & Technology developed a simple device that uses a BITalino device coupled with pulse oximetry and temperature sensors to measure these vital signs (Project HomeSafe), however, the cost of such station is still high. Our novel specialized low-cost wearable is open-source, easily upgradable, and unlike the previously mentioned alternatives, uses an IR thermometer, reducing the overall acquisition time; furthermore, all the sensors are integrated in a single device. Despite the latest advances in telemedicine and mHealth, existing systems are still hindered by high deployment costs, proprietary protocols, closed hardware and software, intrusive form factors, and/or long learning curves. We offer an open-source solution that can be easily upgraded and integrated into other systems. The cloud platform also offers an easy way to visualize data in a user-friendly website.

## 4. Proposed Approach

This section describes the main components of the proposed flexible system. It is made up of three main blocks, designed to function as a whole, but each block can also work separately and independently.

### 4.1. Overall Architecture

The e-CoVig system is a low-cost mHealth solution, coupling a cloud-based e-Health platform for the collection, management, and visualization of physiological and clinical data to a ubiquitous system for acquiring symptomatic measures, using the mobile phone as the primary interface. It seeks to automate the process of follow-up and monitoring of a large number of patients. Monitored parameters include dense recording of temperature, oxygen saturation (${\mathrm{SpO}}_{2}$), photoplethysmography (PPG), heart rate (HR), audio (for the characterization of the cough and respiration), as well as self-report questionnaires with COVID-19 symptomatology or other relevant clinical data for the characterization of the pathology.

**Figure 2:**
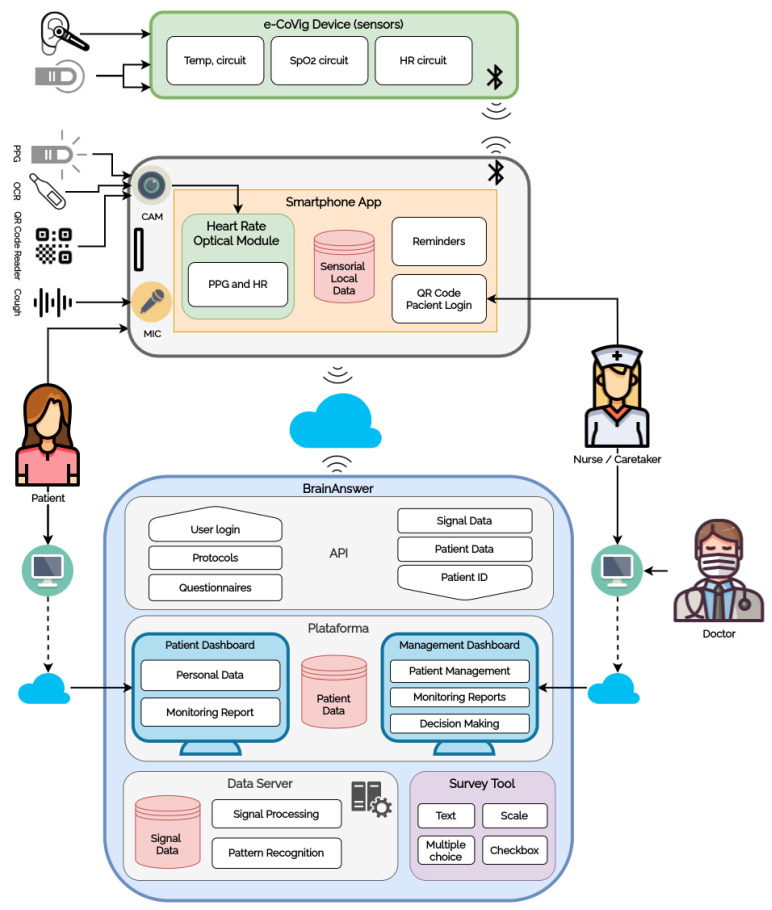
Detailed e-CoVig mHealth system architecture.

Physiological measurements, namely ${\mathrm{SpO}}_{2}$, HR, and temperature are collected using a low-cost and easy-to-use specialized device. The BrainAnswer data visualization and monitoring platform (BrainAnswer—Neuroscience in your hands. https://brainanswer.pt/, accessed on 20 March 2021) is used as cloud-base storage, analytics engine, and patient and healthcare professional dashboards portal. e-CoVig is intended to facilitate the automated interaction between the user and the national healthcare systems, contributing to reduce the risk of contamination and densifying the monitoring process over time, by increasing the number of symptomatic measures. The system can also be extended to other units in the public, private, or social sector. Figure 2 presents the detailed architecture of the system.

### 4.2. Session Management and Authentication

Individuals undergoing e-CoVig monitoring are assigned a unique QR code that can be used across the different components of the system (i.e., mobile and web apps). The mobile app is installed either by the patient, informal carer, or healthcare professional and serves as the primary data collection interface.

**Figure 3:**
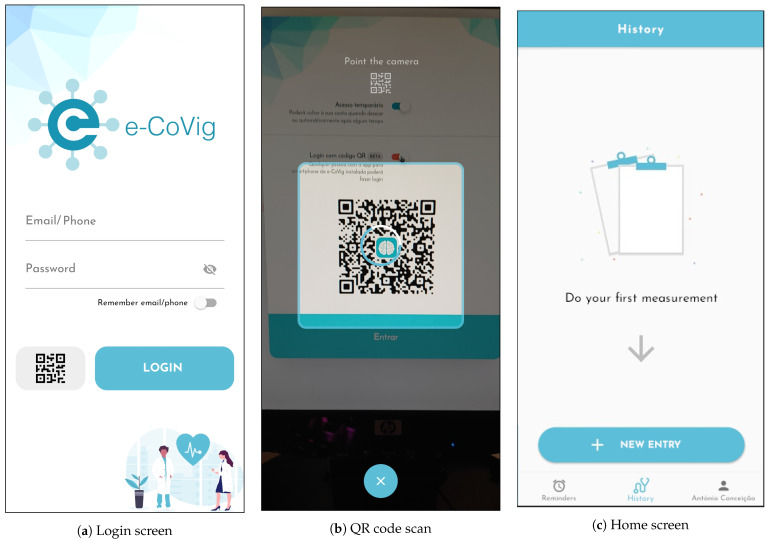
e-CoVig login options and home screen.

When the user opens the application for the first time, the app opens the Login Screen (Figure 3a), where it is possible to sign in to the application with login credentials email/phone number, and password, or by scanning the previously generated personal QR code (Figure 3b). After successfully signing in, the user unlocks the application features (Figure 3c).

The QR code is particularly useful to simplify the login process for patients less proficient in smartphone use. It also enables batch processing of multiple patients in internment or in institutions, e.g., elderly homes, correctional facilities, and others in which it may be of added value to monitor groups of patients sequentially.

All the data collected with the mobile app are stored locally and synchronized with the BrainAnswer cloud back-end whenever an Internet connection is available. BrainAnswer also provides a web-app with a user-friendly analytics dashboard, through which healthcare professionals can review the status of the population that they are following, receive alerts for patients in which abnormal parameters are detected (e.g., hypoxia, high fever), and review the collected qualitative and quantitative data.

### 4.3. Mobile App

The mobile application is the preferred component of the system for the acquisition and transmission of the data for the subjects being monitored. Physiological and clinical data are acquired and sent to the cloud platform through this smartphone-based platform, where the healthcare personnel can follow the symptoms’ evolution and general health of the subjects. Its main functionalities are as follows:

- Electronic Diary (e-Diary)

Symptoms are periodically assessed using an in-app patient electronic diary; self-reporting questionnaires enable the recording of other relevant clinical data to support the pathology characterization. These questionnaires divide symptoms into two categories: moderate and severe. In the moderate section, patients can report symptoms such as mild fever, cough, headaches, muscle pain, weakness, loss of smell or taste, and sore throat. In the severe section, the symptoms monitored are high fever (>38 °C), shortness of breath, peripheral cyanosis, chest pain, loss of consciousness, bloody cough, and vomiting or diarrhea. Taking into account that COVID-19 can have a heavy toll on patients’ overall well-being, the questionnaire includes questions regarding anxiety, quality of sleep, focusing state, and overall feeling.

**Figure 4:**
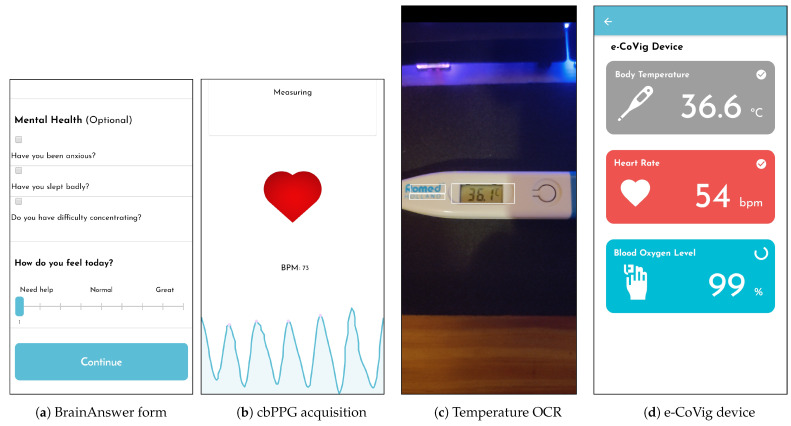
Examples of qualitative and quantitative assessment using e-CoVig.

The patient symptoms diary is implemented using forms (Figure 4a), which can be dynamically and remotely modified through the BrainAnswer cloud platform by health professionals (Figure 2), to better suit each patients’ needs and provide a simple way to gather information regarding the patient and his symptoms.

Quantitative assessment of physiological parameters can be performed momentarily or near-continuously with the specialized device (when applicable). The mobile app also issues reminders and enables the patients to access historical data. Physiological measurements can be performed through multiple methods, as described next.

- Optical Character Recognition (OCR)

Using the camera, the mobile application is capable of performing optical character recognition (OCR) on pictures taken to a digital display. This technique allows the patient to automatically retrieve text from household devices that might be available using the camera feed. In our system, this feature is used to retrieve the temperature value from thermometers (Figure 4c), or the blood oxygenation value from pulse oximeters (POxs). This feature can be extended to acquire the blood pressure values from a blood pressure monitor.

- Respiration and Cough Recording

Using the microphone on the smartphone, it is possible to record the patients’ respiration or coughing and instantly share it with the health professional. This feature allows for a characterization of the breathing sounds and an assessment of the cough, which is typically hard to describe by the patient itself. The duration of the recording can be adjusted on the web platform to better suit the patient’s needs.

- Camera-based Photoplethysmography (cbPPG)

The application implements a camera-based photoplethysmography (cbPPG) feature, by using the device camera and flash [23,24,25,26,27]. This works on the principle that the blood absorbs irradiated light more than tissues [28,29,30] and, consequently, the blood flow affects the amount of light reflected. By measuring the reflected light, the difference in blood flow volume caused by the cardiovascular dynamics can be detected, Figure 4b.

- BITalino Integration

For patients with enhanced risk or who have already been diagnosed, it is important to measure temperature and ${\mathrm{SpO}}_{2}$, which are not easily obtained accurately using a standard smartphone. To overcome this problem, our application can record such data from BITalino, a commercial device that has been specifically designed to deal with the requirements of multi-modal physiological data acquisition [31]. This also allows the acquisition of other sensors that can be interfaced with a BITalino device, such as electrocardiography (ECG) signals, which can provide valuable cardiovascular information.

- e-CoVig Device

Due to the cabled connections of the sensors in the BITalino device and its cost compared to standard digital thermometers and POxs, we developed a specialized low-cost device (see Section 4.4). It has all the sensors integrated into an ergonomic form factor, wireless connectivity, and is equipped with specific sensors of interest in the remote monitoring of COVID-19. The device is assigned to the patient, allowing the recording of body temperature, heart rate, and ${\mathrm{SpO}}_{2}$. The application is capable of communicating with the developed device and retrieving the measurements from the onboard sensors (Figure 4d).

### 4.4. Specialized Device

**Figure 5:**
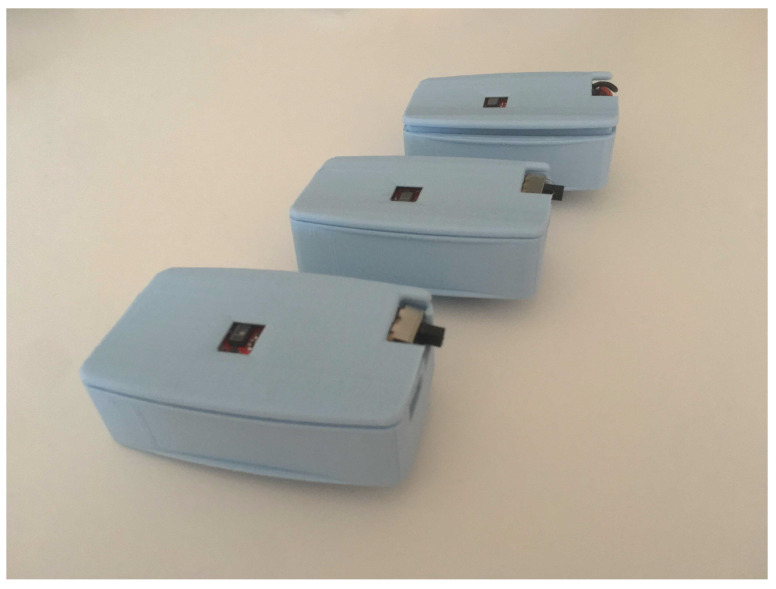
Photo of the developed specialized device.

A specialized device was built using the low-cost and low-power system ESP32 microcontroller (Espressif Systems, Shanghai, China) coupled with specialized sensors (Figure 5). The ESP32 is based on a single-core 32-bit microprocessor running at 160 MHz, 520 KiB of static RAM, integrating Wi-Fi and Bluetooth (classic and BLE). It can be powered using a small LiPo battery.

**Figure 6:**
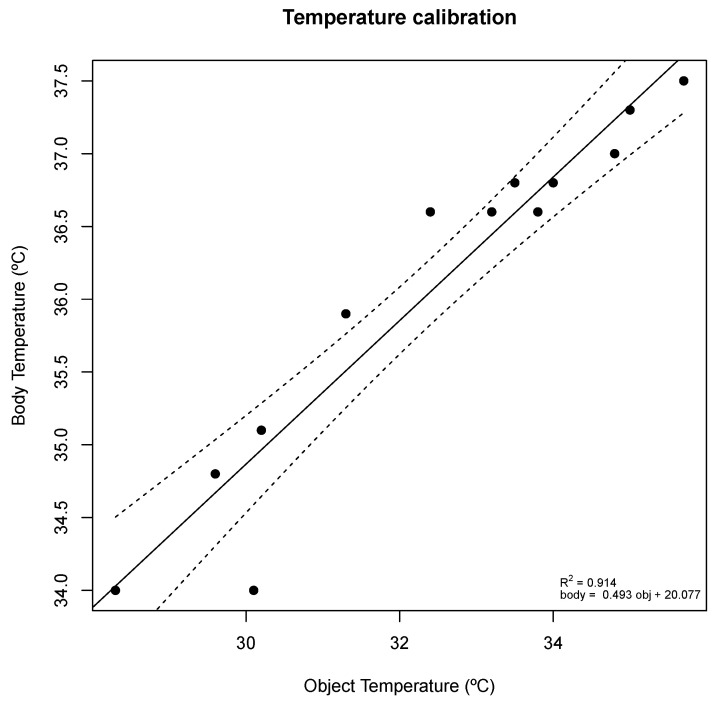
Object and body temperatures measured with a F102 IR thermometer. A linear regression was computed to obtain the linear transformation performed between both temperatures.

The device, specialized in ${\mathrm{SpO}}_{2}$ and temperature acquisition, uses the following sensors:

- Maxim Integrated MAX30101 (Maxim Integrated, San Jose, CA, USA) high-sensitivity pulse oximeter and heart-rate sensor for wearable health, coupled with the MAX32664 biometric sensor hub (Maxim Integrated, San Jose, CA, USA). Once a finger covers the MAX30101 sensor, light-dependent PPG signals are captured and filtered. These signals are passed to the MAX32664 sensor hub, which uses the available information to compute the blood oxygenation and heart rate. The computed values are transmitted to the ESP32 using I2C serial communication protocol.
- Melexis MLX90614 digital non-contact infrared thermometer (Melexis, Ieper, Belgium). The sensor measures the surface temperature of any object is pointing to; temperature can be measured by pointing the sensor to a relevant body part, e.g., forehead skin or inner ear (tympanic temperature). The ESP32 queries the temperature sensor using I2C. The thermometer measures the object (surface) temperature, however, the forehead skin temperature does not correspond directly to the subject’s core body temperature. Common IR thermometers convert the object temperature into body temperature. It was assumed that this transformation follows a linear regression. Object and body pair temperature measurements were taken with an F102 IR thermometer to obtain a linear equation that translates forehead object temperature measurements into body temperature estimations. The results are displayed in Figure 6.

The device is constantly gathering these three measurements (${\mathrm{SpO}}_{2}$, heart rate, and temperature) and, once a Bluetooth connection is established, the values are sent via Bluetooth Serial in a JSON format to the smartphone, as shown below:


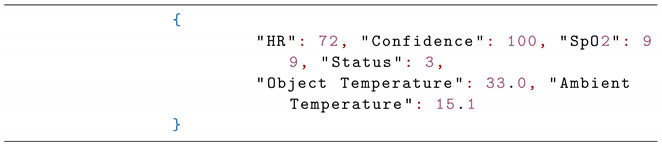


The *Confidence* variable represents the confidence of the ${\mathrm{SpO}}_{2}$ sensor, *Status* reflects the status of the ${\mathrm{SpO}}_{2}$ sensor (0: Success, 1: Not Ready, 2: Object Detected, 3: Finger Detected), and the remaining are self-explanatory. A 3D-printed case was designed to encapsulate the developed device.

The measurement of body temperature at the forehead may lack precision, especially since it is more susceptible to acquisition conditions variability, e.g., the distance from the sensor to the skin greatly impacts the measured temperature. A more accurate and reliable approach would be measuring the tympanic temperature using the IR thermometer, which is a better representation of the subject’s body temperature, therefore, not requiring a linear transformation. The device takes periodic measures of temperature evenly spaced by 0.5 s and, to avoid small fluctuations, a moving average filter of width 3 is applied.

### 4.5. Workflow

Although our approach can be applied to other use cases, it has been designed for mild or asymptomatic patients at home or in isolated accommodations. It aims to provide an easy and automated method to register symptoms and vital signs and share them with the appropriate healthcare professional.

In case of COVID-19 suspicion, the patient contacts or is referenced by the healthcare professional, which then creates an e-CoVig account. After receiving the credentials or personal QR code, the patient completes his/her profile on the BrainAnswer platform, where the remote monitoring procedure is explained. The patient then installs the application on the smartphone (e-CoVig—available on Google Play. https://play.google.com/store/apps/details?id=com.ecovig.app, accessed on 20 March 2021).

In more severe cases, but still, of home quarantine, an e-CoVig device is either delivered at the healthcare facility or mailed to the patient’s address. Within the smartphone application, the patient has a specific measurement protocol that he should follow. Instructions on how to use the device and platform are provided to the patient using the device by the responsible healthcare professional to assure good conditions for the acquisition of vital signs. Video instructions for self-guided operation are also provided (https://brainanswer.pt/website/project/ecovig/video-ecovig.mp4, accessed on 20 March 2021).

**Figure 7:**
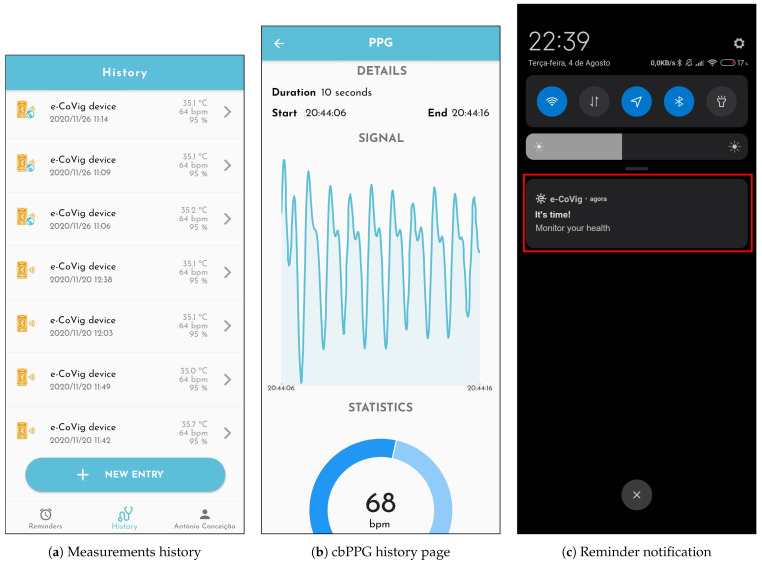
Measurement, history, and reminders

The application can store all the collected information locally (Figure 7a), enabling the patient to review the history data (e.g., Figure 7b). It can also send the information to the cloud-based BrainAnswer platform, through which it can be shared with health professionals. The BrainAnswer platform also integrates algorithms for feature extraction and risk assessment of the monitored subjects, following the guidelines set forth by the COVID-19 management task force in Portugal.

Reminders can also be defined (Figure 7c) so that the patient never forgets to measure his vital signs and share them with healthcare professionals. These reminders can be defined by the patients and healthcare professionals previously, or remotely according to the patient’s medical needs.

On the BrainAnswer platform side, a healthcare professional is responsible for the follow-up of a given patient (or a group of patients). The professional can visualize on his dashboard the information submitted by the various patients. If they suspect that some patient is at greater risk, it is possible to remotely set a stricter acquisition regime for the patient or to contact him via the contacts provided during registration. This way, a single health professional could manage a larger number of patients, decreasing the gathering of people in health centers, and making more efficient the work of medical helplines.

## 5. Results

This section presents the experiments that were done to validate the system especially addressing the acquisition of physiological data.

### 5.1. Participants, Procedure, and Statistics

We designed a cross-sectional study to ascertain the reproducibility and accuracy of the measurements of ${\mathrm{SpO}}_{2}$, heart rate, and temperature with the e-CoVig device. A priori sample size was estimated with the G*Power software, version 3.1.9.6 (Universität Kiel, Germany) [32,33], for a medium-size effect, a power of 0.80, and an error probability of 0.05, resulting in a minimum sample of 27 participants to achieve the criteria. Accordingly, thirty volunteers were recruited, by convenience, among the students and staff of Coimbra’s Polytechnic Institute to participate in the study. The main characteristics of the population are summarized in Table 1.

Written informed consent from each participant was obtained, and all data were treated confidentially and anonymously. The Ethical Commission of the Polytechnic Institute of Coimbra approved the study.

All evaluations were performed in the morning, in a laboratory with appropriate conditions and controlled humidity, temperature, light, and sound, with the participant comfortably seated and after a 10 min resting period. Relevant demographic and clinical data were initially collected. For each physiological parameter (${\mathrm{SpO}}_{2}$, heart rate, and temporal and tympanic temperature), four consecutive measurements were taken with an interval of 2 min between measurements, alternating the e-CoVig device (two measurements) with a standard clinically validated device (two measurements).

The reference devices used for comparison were the MX430 Philips Intellivue Patient Monitor (Royal Phillips, Amsterdam, Netherlands) for ${\mathrm{SpO}}_{2}$ and heart rate, the Microlife NC150 non-contact thermometer (Microlife AG Swiss Corporation, Widnau, Switzerland) for the forehead (temporal) temperature, and the Omron GT 510 oto-thermometer (Omron Corporation, Kyoto, Japan) for the ear (tympanic) temperature. The mean value for each pair of measurements obtained with the reference and the e-CoVig device was used for comparison, assessing the degree of agreement between methods. The individual measurements obtained with the e-Covig device were further used for assessing reproducibility.

The data were computerized and processed with R (version 4.0.3). Simple descriptive statistics were applied for demographic and clinical characterization. Data are presented as mean ± standard deviation (SD) for continuous variables, and as frequency (%) for categorical variables. Comparisons between independent groups were performed with Student’s *t*-test, and for repeated measures with Student’s paired *t*-test. The bivariate correlation analysis was performed with Pearson’s R and regression analysis. The criterion for statistical significance used was a *p*-value ≤ 0.05 to a 95% confidence interval.

### 5.2. Reproducibility Results

To study the reproducibility of the e-CoVig device, a repeated-measurements comparison was performed, for which results are summarized in Table 2. As demonstrated, no significant differences were observed for heart rate, oxygen saturation, and temporal and tympanic temperatures. The lower *p*-value of the temperature measures can be explained by the small range of measured values and the precision of the IR sensor used in the specialized e-CoVig device, which is 0.5 °C in this temperature range.

**Figure 8:**
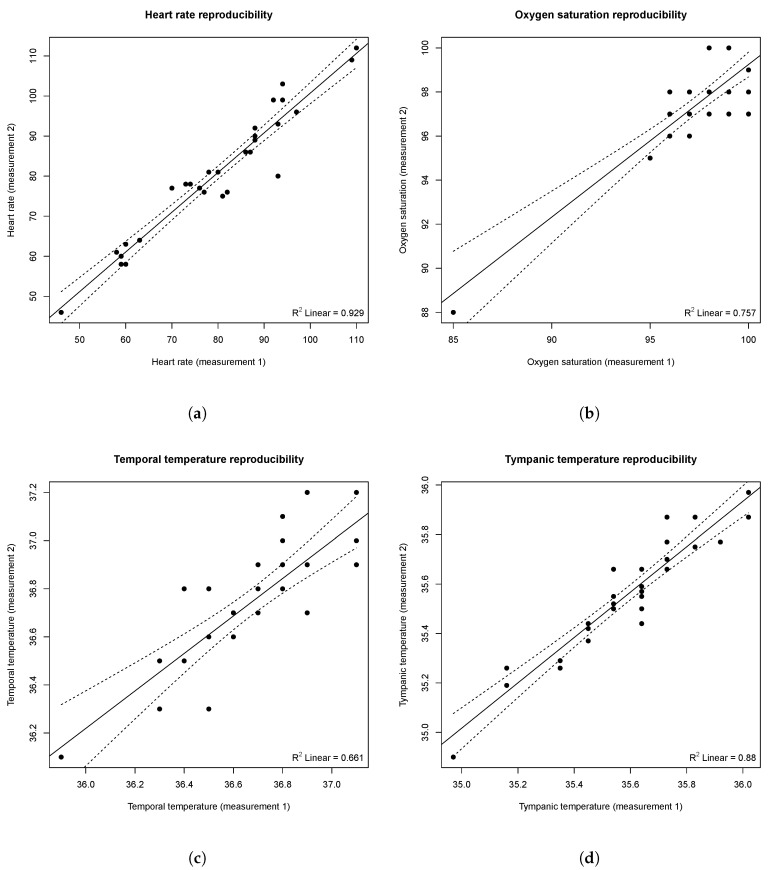
Regression plots representing the e-CoVig repeated-measurements correlation. (**a**) heart rate (in BPM); (**b**) ${\mathrm{SpO}}_{2}$ (in %); (**c**) temporal and (**d**) tympanic temperatures (in °C). The solid line represents the regression line computed using unweighted least squares, while the dashed lines correspond to 95% confidence bands, depicting the upper and lower confidence bounds for all points on the fitted line.

Figure 8 depicts the regression plots for the repeated-measurements with the e-CoVig device, highlighting the significant correlation between measurements buttressed by significant correlation terms for all the physiological variables studied.

### 5.3. Agreement Results

Regarding the comparison between the measurements made with the e-CoVig and the standard clinically validated devices, Table 3 summarizes the pairwise analysis. The mean difference obtained for the e-CoVig tympanic temperature measurement is greater than the temporal temperature (−1.04 ± 0.26 vs. 0.34 ± 0.34, respectively, *p* < 0.001), which can be explained by the fact that the temporal measurements correspond to an estimation of the core temperature from the temperature measured on the forehead skin, while the tympanic temperature is a better representation of the core temperature, not requiring a previous calibration. It is noteworthy that the standard deviation was smaller for the tympanic measurements, indicating that this method of acquisition is less susceptible to variability caused by the acquisition procedure.

**Figure 9:**
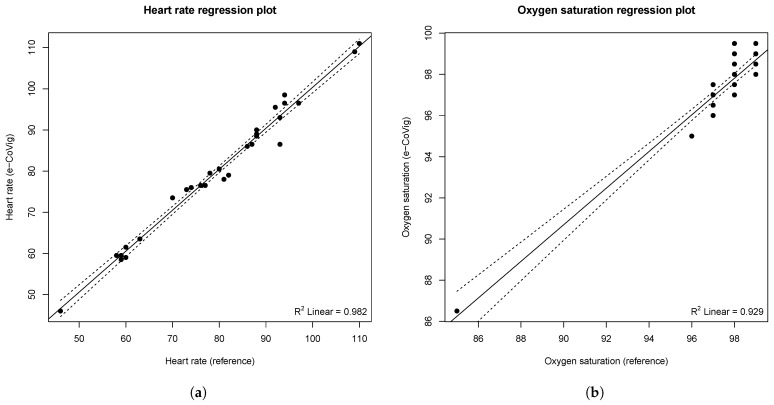

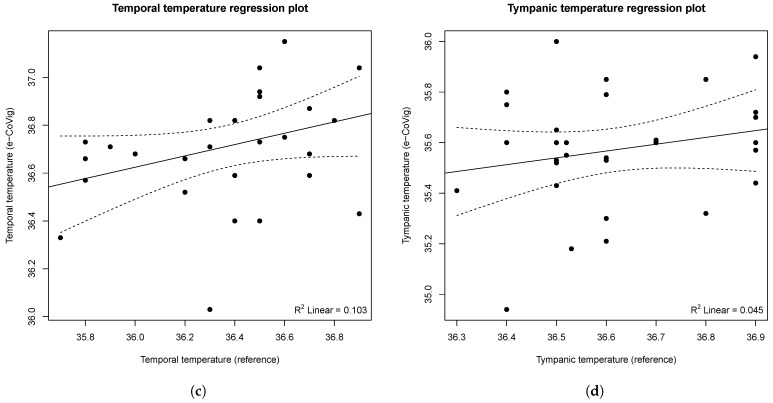
Regression plots representing the correlation between the measurements with the e-CoVig device and the standard reference device. (**a**) heart rate (in BPM); (**b**) ${\mathrm{SpO}}_{2}$ (in %); (**c**) temporal and (**d**) tympanic temperatures (in °C). The solid line represents the regression line computed using unweighted least squares, while the dashed lines correspond to 95% confidence bands, depicting the upper and lower confidence bounds for all points on the fitted line.

A very strong positive and linear correlation was found between the e-CoVig and the standard devices regarding the measurements of heart rate (R = 0.991, *p* < 0.001) and oxygen saturation (R = 0.964, *p* < 0.001). No significant association was found regarding the measurements of temporal (R = 0.322, *p* = 0.083) and tympanic (r = 0.212, *p* = 0.260) temperatures, as depicted in Figure 9. These results are expected, given that the range of temperature measurements is very narrow, less than 1 °C for the tympanic reference temperature, and the IR thermometer has a precision of 0.5 °C within this range. Small variations of the distance between the sensor and the body could have also affected these results. It is speculated that the lower *p*-value of the Pearson’s coefficient for the temporal measurements is due to the wider range of measured temperatures when compared with the tympanic ones (SD: 0.33 vs. 0.18 °C).

## 6. Discussion

The COVID-19 pandemic forced several thousands of people to be confined to their homes. In case of suspicion or confirmation of infection, it is important to monitor the symptoms and vital signs of the subject. Our approach facilitates this monitoring, by providing a mobile-cloud platform that allows patients to record their symptoms, and a specialized device to measure and record physiological signals.

The mobile platform uses a cloud back-end provided by BrainAnswer, resulting in greater flexibility and modularity of the monitoring procedures. The cloud platform also works as a web portal, which medical professionals can use to visualize and manage their patients’ medical data. The mobile and web platforms were used at Santa Casa da Misericórdia’s Nursing Home of Castelo Branco for monitoring the residents, showing good preliminary results. The platforms were handled by instructed healthcare professionals of this establishment and were a valuable tool to digitize the patients’ data. A total of 30 residents participated and 865 acquisitions were performed during one month of use. The main feature used was the self-reports, which allowed the nursing home to easily store medical information and later visualize it in an organized way. The previous method for storing these data was using paper records, therefore, the e-CoVig platform facilitates the effortless transition to digital records. Promising results were achieved, as there was good adherence, lack of resistance, and nurses reported that using e-CoVig made it easier to store data and share it with other healthcare professionals between shifts.

The smartphone-based methods for recording vital signals, either by using OCR or cbPPG, provide a more objective method of recording important medical data, without requiring the acquisition of extra devices. This enables widespread deployment at a large scale. The cbPPG may also be used for estimating the respiration rate, the stress level, and even the blood oxygenation [24,25,27], which can help detect and prevent cases of silent hypoxia [11].

The developed specialized device presented good results overall, and is integrated with the mobile application, thus enabling the measurement of body temperature, heart rate, and ${\mathrm{SpO}}_{2}$. These three vital signals are very important to monitor in COVID-19 patients. The pulse oximeter sensor showed identical results to a standard clinical pulse oximeter. The IR thermometer on the device can be used to measure the tympanic and temporal temperatures, the latter requiring a linear transformation for estimation of the body temperature. The temporal temperature results presented a mean difference of 0.34 °C and standard deviation of 0.34 °C, which is within the precision of the thermometer in this range, (i.e., 0.5 °C). These results could be improved even further if a sensor more accurate in the body temperature range is used (e.g., the MLX90614ESF-DCC, -DCH, or -DCI.

It is important to note that measuring the body temperature on the forehead comes with some limitations; in a recent article in IEEE Spectrum, Erik Beall described them [34], highlighting the main limitation of the temporal temperature, which is the fact that it estimates the body temperature from the skin temperature in the forehead. This measurement may lack precision and, even though it can give a rough estimation of the body core temperature, an acquisition site more representative of the internal temperature is desirable (e.g., the tympanic temperature as suggested in our approach).

Regarding the tympanic temperature measurements, the results presented a lower standard deviation of differences, while showing a larger mean difference, which could be corrected by adding an offset to the estimation. When measuring the tympanic temperature, a probe cap could be used to standardize the acquisition method, reducing acquisition variability and improving the results. It is important to acknowledge that the *p*-values of the temperature measurements did not present significant values, which may be caused by the limited range of measured temperatures since all the subjects had body temperature values within the normal range. Nevertheless, this is a fundamental first step to ensure that it is worthwhile enrolling real patients in the experimental evaluation. Testing the IR temperature sensor on feverish subjects would provide a wider range of temperatures, further refining the results. Future work should also include the implementation of feedback channels between the medical professional and the patient, so patients can feel safely monitored.

Beside the specialized device, the BITalino device was also integrated into the e-CoVig application, allowing the acquisition of additional physiological signals using this established device; this makes it possible for the device to read ECG (e.g.), enabling the use of e-CoVig not only for monitoring COVID-19 patients, but also cardiac and other chronic patients.

The next step is a full evaluation of the system in a real-world setting. e-CoVig will be deployed for long-term monitoring at least two medical wards where COVID-19 and non-COVID-19 patients are present. In these wards, patients are already monitored using standard clinical equipment, and followed by practitioners who investigate patient symptoms. Concurrent data acquisition using e-CoVig and standard medical practices will enable the validation of the ${\mathrm{SpO}}_{2}$, heart rate, and temperature measurements. It will also establish the base for the development of patient outcomes prediction and modeling, allowing for early intervention from healthcare practitioners.

Overall, e-CoVig showed very good results both in the laboratory and real-world settings. The suggested remote-monitoring paradigm could allow the monitoring of a larger number of patients, without greatly increasing the work overhead for medical professionals and is ready for deployment. This tool can be used for individual monitoring, in the case of subjects under home quarantine, or for collective monitoring, as it is being used to follow-up the residents in a nursing home.

## Figures and Tables

**Table 1:** Main characteristics of the population.

	**Mean**	**Standard Deviation**
Age (years)	23.33	9.67
Height (m)	1.66	0.09
Weight (Kg)	63.37	14.14
Temperature (°C)	36.50	0.24
	**N**	**%**
Gender (female)	22	73.30
Smoking habits (yes)	1	3.30
Sedentary (yes)	14	46.70
Arterial hypertension (yes)	0	0.00
Dyslipidaemia (yes)	2	6.70
Diabetes (yes)	0	0.00
Other clinical conditions (yes)	0	0.00
Medication (yes)	2	6.70

**Table 2:** Pairwise comparison of the e-CoVig repeated measurements.

	1st Measurement	2nd Measurement	Mean Difference	*p*-Value
Heart rate (BPM)	80.10±15.60	81.00±16.00	0.90±4.30	0.257
${\mathrm{SpO}}_{2}$ (%)	97.30±2.60	97.40±2.10	0.10±1.30	0.783
Temporal temperature (°C)	36.68±0.26	36.75±0.25	0.07±0.16	0.020
Tympanic temperature (°C)	35.59±0.24	35.56±0.23	−0.03±0.08	0.045

**Table 3:** Pairwise comparison between the e-CoVig and the standard device measurements.

	Standard Device	e-CoVig Device	Mean Difference	*p*-Value
Heart rate (BPM)	80.10±15.60	80.50±15.70	0.40±2.10	0.912
${\mathrm{SpO}}_{2}$ (%)	97.50±2.50	97.40±2.30	−0.10±0.70	0.829
Temporal temperature (°C)	36.37±0.33	36.71±0.24	0.34±0.34	<0.001
Tympanic temperature (°C)	36.62±0.18	35.57±0.23	−1.04±0.26	<0.001

## Data Availability

The data presented in this study are available upon reasonable request made to the corresponding author. The data are not publicly available due to privacy restrictions.
